# An Investigation into the Stability Source of Collagen Fiber Modified Using Cr(III): An Adsorption Isotherm Study

**DOI:** 10.3390/molecules29020300

**Published:** 2024-01-06

**Authors:** Jiheng Li, Wenjun Long, Liangqiong Peng, Lijun Guo, Wenhua Zhang

**Affiliations:** 1 Key Laboratory of Leather Chemistry and Engineering of Ministry of Education, Sichuan University, Chengdu 610065, China; 2021223080023@stu.scu.edu.cn; 2 National Engineering Laboratory for Clean Technology of Leather Manufacture, Sichuan University, Chengdu 610065, China

**Keywords:** chrome leather, stability mechanism, adsorption thermodynamics, entropy

## Abstract

The enhanced hydrothermal stability of leather, imparted by little Cr(III), has traditionally been ascribed to strong coordinate bonds. However, this explanation falls short when considering that the heat-induced shrinking of collagen fiber is predominantly driven by rupturing weak H-bonds. This study explored the stability source via adsorption thermodynamics using collagen fiber as an adsorbent. Eleven isotherm models were fitted with the equilibrium dataset. Nine of these models aptly described Cr(III) adsorption based on the physical interpretations of model parameters and error functions. The adsorption equilibrium constants from six models could be transformed into dimensionless thermodynamic equilibrium constants. Based on the higher *R*^2^ of the van’t Hoff equation, thermodynamic parameters (∆*G*°, ∆*H*°, ∆*S*°) from the Fritz–Shluender isotherm model revealed that the adsorption process typifies endothermic and spontaneous chemisorption, emphasizing entropy increase as the primary driver of Cr(III) bonding with collagen. Thus, the release of bound H_2_O from collagen is identified as the stability source of collagen fiber modified by Cr(III). This research not only clarifies the selection and applicability of the isotherm model in a specific aqueous system but also identifies entropy, rather than enthalpy, as the principal stability source of Cr-leather. These insights facilitate the development of novel methods to obtain stable collagen-based material.

## 1. Introduction

Putrescible animal skins can be readily converted into hydrothermally stable collagen material using a minute amount of Cr(III) to facilitate cross-linking between collagen molecules. This method has been widely employed in the leather industry as Cr(III) tanning to produce over 80% of leather products [1,2]. For instance, using only 1.8 wt.% Cr(III) of the skin mass can significantly increase the shrinkage temperature (Ts) of the material from 51 °C to 90 °C [3]. This implies that intermolecular cross-linking of all 140 amino acids along the collagen peptide chain can considerably enhance thermal stability, especially considering that about 70% of Cr(III) can bond with the collagen sidechain. Of this, only 20% of polymeric three-center and four-center Cr(III) effectively cross-links adjacent collagen molecules [4]. This phenomenon has piqued significant interest in understanding its source of hydrothermal stability.

The dominant stability was initially attributed to the robust coordinate interactions between polynuclear Cr(III) and ionized carboxyl groups of collagen side chains. However, during the heat shrinking of collagen fibers, these coordinate bonds remain intact [5]. The thermodynamics of the shrinking process primarily indicates H-bond breakdown, irrespective of whether the cross-linking bond is coordination or covalence. The link-lock theory was subsequently proposed, suggesting that crosslinkers such as Cr(III) influence the size of the cooperating unit, with larger units corresponding to higher Ts [6]. Nevertheless, the nature of the cooperating unit remains unclear. The cross-linking reaction is intricate at the molecular level, necessitating a deeper exploration into the essence of hydrothermal stability. This understanding is pivotal for developing cleaner and more efficient cross-linking processes to produce diverse collagen-based functional materials.

The hierarchical structure of collagen fibers offers a high micropore volume and low mass transfer resistance. Such fibers have been utilized as adsorbents for removing aqueous heavy metals [7] and organic contaminants [8]. In the cross-linking process of collagen fiber with Cr(III), aqueous Cr(III) ions are drawn to the surface of collagen fibers through physical and chemical interactions, similar to the liquid–solid adsorption process. Understanding adsorption kinetics and equilibrium is vital for a thorough comprehension of the cross-linking process. Specifically, isotherm models are often employed to elucidate adsorption mechanisms, and various adsorption isotherms have been devised to uncover lesser-known facets of the adsorption process [9]. Furthermore, adsorption thermodynamics is crucial for determining whether the mechanism is physical or chemical. Thermodynamic parameters (standard Gibbs free energy change ∆*G*°, standard enthalpy change ∆*H*°, and standard entropy change ∆*S*°) have been both directly and indirectly calculated using equilibrium constants or constants from corresponding adsorption isotherm models [10]. Several researchers have reviewed isotherm models suitable for liquid-phase adsorption processes, offering insights into fitting methods, error functions, underlying assumptions, and practical applications [11,12].

This study utilized collagen fibers as an adsorbent to investigate the stability source of a collagen material modified by Cr(III) through adsorption equilibrium and thermodynamics. The effects of solution pH and temperature were systematically investigated. Various isotherm models, including Langmuir, Freundlich, Halsey, Brouers–Sotolongo, Fritz–Shluender, Koble–Corrigan, Radke–Prausnitz, Redlich–Peterson, Sips, and Vieth–Sladek, were employed to fit the experimental adsorption isotherm data. Thermodynamic parameters were determined from the various model constants and further analyzed. This work not only offers a comprehensive explanation and illustration of the application criteria of isotherm models, facilitating the advancement of adsorption theory but also aids in developing innovative approaches to stabilize collagen material.

## 2. Results and Discussion

### 2.1. Adsorption Isotherm of Cr(III) onto Collagen Fibers

**Figure 1:**
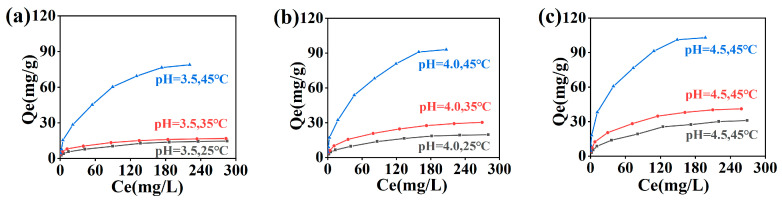
Adsorption isotherm of Cr(III) at pH 3.5 (**a**) (black line), 4.0 (**b**) (red line), and 4.5 (**c**) (blue line).

The adsorption of Cr(III) onto collagen fibers, at varying solution pH and temperatures, reached equilibrium within 12 h, as depicted in Figure 1. The isotherm of Cr(III) adsorption onto collagen fibers under different solution pH levels and temperatures is presented in Figure 1. Both temperature and initial solution pH had notable effects on the adsorption behavior of the collagen fiber for Cr(III). With increasing temperature at each solution pH, the adsorption capacity for Cr(III) also rose. A marked increase was observed at 45 °C. For instance, in a solution with an initial pH of 4.0, the adsorption capacity of Cr(III) increased from 19.65 mg/g to 30.34 mg/g when the temperature increased from 25 °C to 35 °C. This value then surged to 93.04 mg/g at 45 °C. Concurrently, the adsorption capacity for Cr(III) is augmented with the rise in initial solution pH at each respective temperature. For instance, at 35 °C, the adsorption capacity for Cr(III) increased from 16.88 mg/g to 30.34 mg/g with a pH increase from 3.5 to 4.0, further rising to 41.17 mg/g at pH 4.5.

The reaction of Cr(III) with collagen fiber is a standard procedure in leather manufacturing due to the high thermal stability of chrome leather. The reaction process typically modulates solution pH and temperature profiles to manage the balance between the penetration and fixation of Cr(III) through pelts with limited thickness [1]. Initially, both the solution pH and temperature are set to around 3.0 and below 30 °C, respectively. In this environment, the Cr(III) species of smaller size dominate (as seen in Figure S2), which aligns with the reduced availability of ionized carboxyl groups in collagen. This ensures a uniform penetration of Cr(III). Subsequent elevation in solution pH and temperature enhances the proportion of ionized carboxyls and polymerized chrome species. This, in turn, catalyzes the formation of covalent complexes between the Cr(III) species and collagen carboxyl groups, thereby increasing the shrinkage temperature of the collagen fiber [1]. Thus, investigating the adsorption behavior of collagen fiber for Cr(III) can provide insights into the stability source of chrome tannage technology.

### 2.2. Adsorption Isotherm Fitting

The adsorption isotherm data from Figure 1 were analyzed using 11 selected adsorption isotherm models. Considering the errors associated with the conventional linear method [13], model parameters were determined through nonlinear optimization, as detailed in Table 1. Statistical parameters, such as the coefficient of determination *R*^2^, adjusted *R*^2^, and nonlinear chi-square *χ*^2^, are also provided in Table 1.

All 11 adsorption isotherm models can describe the adsorption process, as evidenced by *R*^2^ values greater than 0.91. Considering the higher *R*^2^ and ajd-*R*^2^ values and lower *χ*^2^ values, the fitting effectiveness of the models can be ranked as follows: Brouers–Sotolongo model ≈ Koble–Corrigan model ≈ Sips model > Jossens model ≈ Redlich–Peterson > Freundlich model ≈ Halsey model > Fritz–Schluender model ≈ Radke–Prausnitz model > Vieth–Sladek model > Langmuir model. Additionally, the applicability of certain models, such as the Koble–Corrigan and Radke–Prausnitz models, should be reconsidered based on their physical interpretations. For instance, the exponent $n_{KC}$ of the Koble–Corrigan model is less than 1, indicating its limitations in accurately representing the experimental data [9,14]. A negative value was also observed for the Radke–Prausnitz model, suggesting constraints when adapting these models from gas–solid phases to liquid–solid adsorption systems. Hence, nine models can be employed to explore the stability mechanisms of collagen fiber endowed by chrome: the Brouers–Sotolongo, Sips, Jossens, Redlich–Peterson, Freundlich, Halsey, Fritz–Schluender, Vieth–Sladek, and Langmuir models.

The optimally fitted Brouers–Sotolongo isotherm model suggests that the collagen fiber possesses active sites with distributed sorption energies for chrome species, consistent with the Levy stable distributions theory [15]. The Sips isotherm supports the properties of monolayer and heterogeneous adsorption [16]. The Jossens isotherm indicates that the surface of the adsorbent is uneven and exhibits multi-layer adsorption. The Redlich–Peterson isotherm is suitable for studying homogeneous and heterogeneous adsorption over a wide concentration range [17]. The Freundlich isotherm confirms the uneven and multisite (heterogeneous) nature of fiber surfaces, while the Halsey isotherm implies multi-layer chrome adsorption, which might arise from the multinuclear chrome species, particularly in higher pH solutions (see Figure S2). The Fritz–Schluender model’s good fit hints at the competitive adsorption of chrome species onto collagen fiber. Moreover, both the linear and nonlinear components of the Vieth–Sladek isotherm model elucidate the physical and chemical adherence of chrome species to amorphous and porous adsorbent surfaces. The Langmuir isotherm model highlights the reversible nature of the chrome adsorption–desorption process and offers an improved prediction of the maximum adsorption capacity. Furthermore, the inferior fit of the Langmuir model compared to the other eight isotherm models supports the concept of heterogeneous and competitive adsorption inherent to chrome tanning.

### 2.3. Adsorption Thermodynamics

The energetic parameters of an adsorption process can be deduced from its associated thermodynamic parameters. Understanding these parameters can provide deeper insights into the underlying mechanism of the process. To obtain these thermodynamic parameters, the thermodynamic equilibrium constant $K_{Eq}^{0}$ should be calculated from the isotherm model equilibrium constant *K_Model_*, as described in Equations (19)–(21). The isotherm model equilibrium constants of the Sips, Jossens, Redlich–Peterson, Fritz–Schluender, Vieth–Sladek, and Langmuir models can be used to calculate $K_{Eq}^{0}$ when considering improved fitting with the experimental data and the dimension of *K_Model_*. Notably, there is no difference in the sign of thermodynamic parameters (∆*H*° and ∆*S*°) when derived from both linear and nonlinear versions of the van’t Hoff equation [18]. Thus, we adopted the linear form to calculate the thermodynamic parameters. To indicate the correlation, the coefficient of determination (*R*^2^) was calculated, as shown in Table 2.

The ∆*G*°’s sign, determined from the six adsorption isotherm models, was consistently negative as per Equation (16), and their magnitudes were also similar. However, the value of ∆*G*° from the Langmuir and Sips models deviated to some extent from the other four models, aligning with its less accurate fit to the experimental equilibrium data, as evidenced by the Langmuir model’s lower *R*^2^ and higher *χ*^2^ values (Table 1). In contrast, the ∆*H*° and ∆*S*° varied in both sign and magnitude, indicating that the calculation of thermodynamic parameters from the isotherm model should be very prudent to reach the correct conclusion. Then, the *R*^2^ of the van’t Hoff equation was calculated to exhibit significant differences among the six isotherm models, indicating a hierarchy in the appropriateness of calculating thermodynamic parameters from the models: Fritz–Shluender model >> Redlich–Peterson >> Jossens >> Sips >> Vieth–Sladek model >> Langmuir model. Therefore, the adsorption thermodynamics of Cr(III) onto collagen fiber should be further discussed based on the Fritz–Schluender model.

A negative ∆*G*° indicates that the adsorption of Cr(III) onto collagen fiber occurs spontaneously, and lower ∆*G*° values suggest that the equilibrium shifts towards adsorption with increasing temperature or solution pH. This supports the chrome tannage technology, which operates at higher pH and temperatures to ensure optimal exhaustion of chrome in the last step [1]. Furthermore, the positive ∆*H*° underscores the endothermic nature of the adsorption process, increasing from 48.43 kJ/mol to 61.36 kJ/mol as the solution pH rises from 3.5 to 4.5. A ∆*H*° magnitude exceeding 40 kJ/mol further indicates the chemisorptive nature of the collagen fiber’s interaction with Cr(III) [19].

The heightened spontaneity (∆*G*°) associated with higher-solution pH is driven by a substantial increase in the system’s entropy. Specifically, the ∆*S*° of the system rose by 45 J‧mol^−1^‧K^−1^ when the solution pH was adjusted from 3.5 to 4.5 at respective three temperatures. Thus, the adsorption of collagen fiber for Cr(III) is primarily driven by the increase in entropy of the process. However, when the Cr(III) species were adsorbed onto the collagen fiber, there was a decrease in translational freedom, which negatively contributed to the entropy change of the system. The entropy increase during the adsorption process might be attributed to the release of bound H_2_O from the collagen into the solution and the concurrent changes in collagen protein conformation. Indeed, Cr(III) was adsorbed on the polar active sites of collagen fiber, which was initially bound with H_2_O via a H-bond. Additionally, when the multinuclear chrome species was co-adsorbed by neighboring parallel collagen molecules, the lateral spacing between collagen molecules was generated and maintained to a certain extent [20], which might lead to the rupture of the H-bond formed via H_2_O and the adjacent polar sidechain of collagen, resulting in more bound H_2_O liberation. This speculation can be validated because the adsorption process was accompanied by a higher entropy increase under a higher-solution pH from the best-fitted Fritz–Schluender model (Table 2). Obviously, the adsorption of Cr(III) would produce a phenomenon similar to the dehydration of collagen fiber. Thus, dehydration was identified as the dominating role of endowing the stability of chrome leather, which was confirmed by the report about novel leather technology by making collagen fibers superhydrophobic [21]. Such insights could clarify the stability mechanisms of collagen materials and pave the way for developing innovative methods for functional collagen materials.

### 2.4. Material Characterization

**Figure 2:**
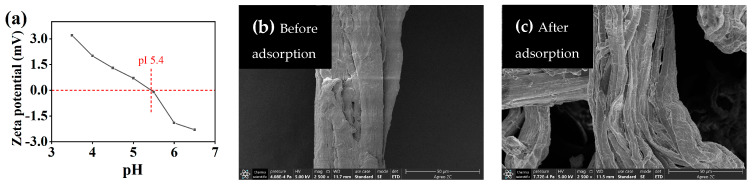
Zeta potential of collagen fibers (**a**) (collagen fiber isoelectric point (pI (red line)) = 5.4), scanning electron microscopy (SEM) image (**b**,**c**) of collagen fiber tanned Cr(III) (2500×, [Cr(III)] = 200 mg/L, T = 308 K, pH 4.0).

Collagen fibers have an isoelectric point of 5.4, as shown in Figure 2a. When the pH value of the solution is lower than its isoelectric point, the surface of collagen fibers charges positively, which is mainly caused by the protonation of –NH_2_ in the sidechain of collagen. Then, the positive Cr(III) mainly binds to the COOH/COO^−^ of side chains on collagen fibers, consistent with previous conclusions [22].

The SEM results (Figure 2b,c) observed that after the adsorption of Cr(III), collagen fibers became more dispersed, indicating a change in the adsorbent structure induced by Cr(III).

**Figure 3:**
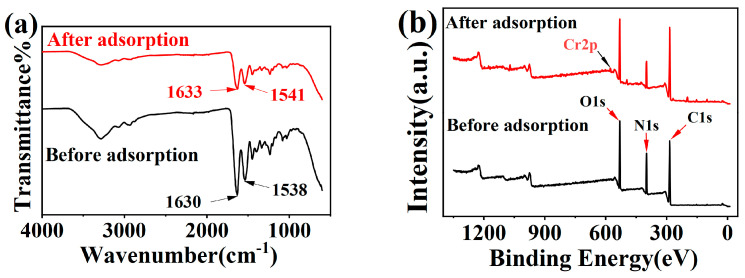

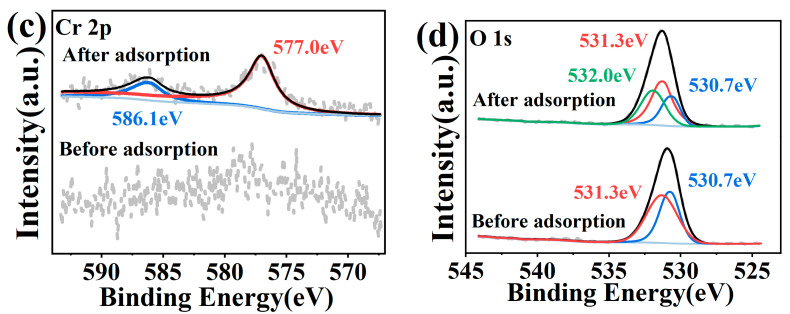
FTIR spectra (**a**) (the black line represents before adsorption, the red line represents after adsorption), XPS spectra of survey scans (**b**) (the black and red lines represent before and after adsorption, respectively), Cr2p (**c**) (blue is the line where the 586.1 eV peak is located, Red is the line where the 577.0 eV peak is located) and O1s (**d**) (red and blue represent C=O and C–O bonds, respectively, green represents the Cr–O bond) ([Cr(III)] = 100 mg/L, pH = 4.5, T = 308 K).

FTIR spectra of collagen fibers before and after adsorption are depicted in Figure 3a. The peak at 1630 cm^−1^, which indicates the deprotonated carboxylate’s asymmetric stretching vibration (COO^−^), shifted to 1633 cm^−1^ after adsorption. This suggests an interaction with Cr(III). This inference is further supported by the blue shift of the symmetric stretching vibration of deprotonated carboxylate (COO^−^) with a peak at 1538 cm^−1^ [23], indicating the binding between collagen fiber’s COO^−^ and Cr(III).

The XPS results are shown in Figure 3b–d. After adsorption, the signal of Cr2p appeared in the XPS spectra of survey scans, indicating that Cr was successfully adsorbed. The two peaks of 577.0 eV and 586.1 eV that appeared in the Cr2p spectra (Figure 3c) are Cr2p_3/2_ and Cr2p_1/2_, respectively [24]. The analysis of the O1s peaks indicates that the peaks at 531.3 and 530.7 eV were C=O and C–O groups, respectively. The new peak appearing at 532.0 eV is attributed to Cr–O bond (chromium–oxygen single bond) [25,26]. This confirms the chemical adsorption of Cr(III) by collagen fibers.

## 3. Materials and Methods

### 3.1. Adsorption Isotherm of Cr(III) onto Collagen Fibers

A quantity of 0.05 g of collagen fibers (self-produced from cattle skin using a standard procedure [27]) was immersed in 50 mL of a Cr(III) solution. The initial Cr(III) concentration was set to 5, 10, 20, 50, 100, 150, 200, 250, and 300 mg/L with solution pH values of 3.5, 4.0, and 4.5, respectively, adjusted by 0.05 mol/L NaHCO_3_ or 0.05 mol/L HCl (FE28 pH meter, Mettler Toledo Instruments Co., Ltd., Columbus, OH, USA). The solution was shaken at 298, 308, and 318 K for 24 h (ZWY-2102C constant temperature oscillator, Shanghai Zhicheng Analytical Instruments Manufacturing Co., Ltd., Shanghai, China). The Cr(III) concentration in solution was detected using inductively coupled plasma atomic emission spectrometry (Optima 8000DV inductively coupled plasma emission spectrometer, PerkinElmer, Waltham, MA, USA). The equilibrium adsorption capacity *q_e_* (mg/g) is calculated as

(1) $$q_{e}=\frac{(C_{0}-C_{e})V}{m}$$

where *C*_0_ and *C_e_* are the Cr(III) concentration in the solution before adsorption and at adsorption equilibrium, respectively, in mg/L; *V* is the volume of the Cr(III) solution, in L; and *m* is the mass of the collagen fiber, in g. All experiments were conducted in three parallel groups, and all reagents were of analytical grade, procured from Kelong Chemicals (Chengdu, China).

### 3.2. Zeta Potential Measurement of Collagen Fibers

Submerge 10 g of collagen fibers in 400 mL of distilled water and use a 0.2 mol/L HCl solution and NaOH solution, respectively, to alter the pH to various gradients. The solution was shaken at 298 K for 0.5 h (ZWY-2102C constant temperature observer, Shanghai Zhicheng Analytical Instruments Manufacturing Co., Ltd., Shanghai, China). Measure the Zeta potential (MütekTM SZP-10, BTG, Augsburg, BAV, Germany) of the sample using a Zeta potentiometer.

### 3.3. Characterization

Collagen fiber was characterized both before and after adsorption. A scanning electron microscope (SEM, Apreo 2C, Thermo Fisher Scientific, Waltham, MA, USA) and infrared spectra (FTIR, Spectrum 3 Fourier transform medium near infrared dual-band infrared light, PerkinElmer) were employed to analyze the functional groups of collagen fiber over a wavenumber range of 4000–400 cm^−1^. X-ray photoelectron spectroscopy (XPS, ESCALAB 250Xi X-ray photoelectron spectrometer, Thermo Fisher Scientific) was used to analyze the elemental composition of the sample.

### 3.4. Adsorption Isotherm Model

Numerous adsorption isotherm models have been developed to fit experimental adsorption equilibrium data. This study utilized commonly used solid–liquid adsorption isotherm models (Equations (2)–(12)) to explore the stability source of classical Cr(III) tanning using collagen fiber as an adsorbent for Cr(III). In Equations (2)–(12), *C_e_* (mg/L) and *q_e_* (mg/g) represent the Cr(III) equilibrium concentration in a solution and on the adsorbent.

The two-parameter adsorption isotherm models include the Langmuir model for uniform surfaces, the Freundlich model for heterogeneous surfaces, and the Halsey model for heterogeneous porous surfaces (Equations (2)–(4)).

(2) $$q_{e}=\frac{Q_{L}K_{L}C_{e}}{1+K_{L}C_{e}}$$

where *Q_L_* (mg/g) is the Langmuir model’s maximal adsorption capacity, and *K_L_* (L/mg) represents the model’s equilibrium constant [28].

(3) $$q_{e}=K_{F}C_{e}^{n_{F}}$$

where *K_F_* (${\left(\mathrm{mg}/g)/(\mathrm{mg}/L\right)}^{n_{F}}$) is the Freundlich model constant and $n_{F}$ is the model exponent [29].

(4) $$q_{e}=e^{-(\ln K_{HL}-\ln C_{e})/n_{HL}}$$

where *K_HL_* (${\left(\mathrm{mg}/g)/(\mathrm{mg}/L\right)}^{n_{HL}}$) is the Halsey model constant and $n_{HL}$ is the model index [30].

The three-parameter isotherm models include the Brouers–Sotolongo, Fritz–Schlunder, Koble–Corrigan, Radke–Prausnitz, Redlich–Peterson, Sips, and Vieth–Sladek models (Equations (5)–(12)).

(5) $$q_{e}=Q_{BS}(1-e^{\left(-K_{BS}C_{e}^{\alpha_{BS}}\right)})$$

where *Q_BS_* (mg/g) is the Brouers–Sotolongo model’s maximal adsorption capacity; *K_BS_* (${\left(\mathrm{mg}/g)/(\mathrm{mg}/L\right)}^{\alpha_{BS}}$) is the model constant; and $\alpha_{BS}$(L/mg) is the model’s equilibrium constant [31].

(6) $$q_{e}=\frac{Q_{FS}K_{FS}C_{e}}{1+Q_{FS}C_{e}^{m_{FS}}}$$

where *Q_FS_* (mg/g) is the Fritz–Schluender model’s maximal adsorption capacity; *K_FS_* (L/mg) is the equilibrium constant of the model; and $m_{FS}$ is the model index [32].

(7) $$q_{e}=\frac{i_{JS}C_{e}}{1+j_{JS}{\left(C_{e}\right)}^{m_{JS}}}$$

where $i_{JS}$ (L/g) and $j_{JS}$ (${\left(L/\mathrm{mg}\right)}^{n_{\left(RP\right)}}$) are constants of the Jossens isotherm model and $m_{JS}$ is the exponent of the Jossens model (range 0–1) [33].

(8) $$q_{e}=\frac{A_{KC}C_{e}^{n_{KC}}}{1+K_{KC}C_{e}^{n_{KC}}}$$

where *A_KC_* ($\left(\mathrm{mg}/g\right)\times {\left(L/\mathrm{mg}\right)}^{n_{KC}}$) is the Koble–Corrigan model constant; *K_KC_* (${\left(L/\mathrm{mg}\right)}^{n_{KC}}$) is the model equilibrium constant; $Q_{KC}=A_{KC}/K_{KC}$ (mg/g) reflects the maximal adsorption capacity; and $n_{KC}$ is the model index [14].

(9) $$q_{e}=\frac{a_{RP}b_{RP}C_{e}^{m_{RP}}}{a_{RP}+b_{RP}C_{e}^{m_{RP}-1}}$$

where $a_{RP}$ (L/g) and $b_{RP}$ (${\left(\mathrm{mg}/g)/(\mathrm{mg}/L\right)}^{m_{RP}}$) are Radke–Prausnitz model constants; the model equilibrium constant *K_RP_* (${\left(L/g\right)}^{1-m_{RP}}$) can be obtained from a/b; and $m_{RP}$ is the index of the model [34].

(10) $$q_{e}=\frac{K_{\left(RP\right)}C_{e}}{1+a_{\left(RP\right)}C_{e}^{n_{RP}}}$$

where *K_(RP)_* (L/g) is the Redlich–Peterson model constant; $a_{\left(RP\right)}$ (${\left(L/\mathrm{mg}\right)}^{n_{\left(RP\right)}}$) is the model’s equilibrium constant; and $n_{\left(RP\right)}$ is the model index. (range 0–1) [35].

(11) $$q_{e}=\frac{Q_{Sips}K_{Sips}C_{e}^{n_{Sips}}}{1+K_{Sips}C_{e}^{n_{Sips}}}$$

where *Q_Sips_* (mg/g) is the Sips adsorption capacity; *K_Sips_* (${\left(L/\mathrm{mg}\right)}^{n_{Sips}}$) is the Sips model’s equilibrium constant; and $n_{Sips}$ is the model index [36].

The Vieth–Sladek isotherm model includes linear and nonlinear parts, which have a wide adaptability:

(12) $$q_{e}=K_{VS}C_{e}+\frac{Q_{VS}\beta_{VS}C_{e}}{1+\beta_{VS}C_{e}}$$

where *Q_VS_* (mg/g) is the model maximum adsorption capacity; *β*^2^ (L/mg) is the equilibrium constant of the model; and *K_VS_* (L/g) is the model constant [37].

### 3.5. Statistical Parameters

To assess the fitting correlation of the isotherm models, statistical parameters *R*^2^, adjusted *R*^2^, and *χ*^2^ are employed. The *R*^2^ and adjusted *R*^2^ values are widely used statistical parameters, with a value closer to 1 indicating superior model–data alignment.

(13) $$R^{2}=\frac{\sum {\left(q_{mean}-q_{cal}\right)}^{2}}{\sum {\left(q_{cal}-q_{mean}\right)}^{2}+\sum {\left(q_{cal}-q_{exp}\right)}^{2}}$$

(14) $$AdjR^{2}=1-(1-R^{2})\frac{\left(N_{exp}-1\right)}{\left(N_{exp}-N_{para}-1\right)}$$

In Equation (13), *q_mean_* is the average value of experimental adsorption capacity, *q_cal_* is the calculated adsorption capacity, and *q_exp_* is the experimental adsorption capacity. In Equation (14), *N_exp_* is the number of data points, and *N_para_* is the number of parameters. The effective range of *R*^2^ and adjusted *R*^2^ are 0–1.

However, the difference in *R*^2^ and adjusted *R*^2^ values among various isotherm models can be minimal, complicating the choice of the optimal model. Hence, additional statistical parameters, such as *χ*^2^, must be calculated for fitness assessment [38]:

(15) $$\chi^{2}=\sum \frac{{\left(q_{exp}-q_{cal}\right)}^{2}}{q_{cal}^{2}}$$

A lower *χ*^2^ value implies that the predicted results of the isotherm model are closer to the experimental results.

### 3.6. Adsorption Thermodynamics

The standard Gibbs free energy variation (∆*G*°) for the adsorption process can be obtained from the dimensionless thermodynamic equilibrium constant $K_{Eq}^{0}$ as

(16) $$\Delta G{}^{\circ}=-RT\ln K_{Eq}^{0}$$

where *R* is the gas constant (8.314 J/(mol‧K)) and *T* (K) is the temperature. When the reaction’s temperature range is narrow, it can be assumed that temperature has no effect on the standard enthalpy variation (∆*H*°), and the van’t Hoff equation can be employed to describe the relationship between ∆*G*°, ∆*H*°, and the standard variation of entropy (∆*S*°) [19]:

(17) ΔG°=ΔH°−TΔS°

Obviously, the thermodynamic equilibrium constant $K_{Eq}^{0}$ of the adsorption process is essential for calculating the thermodynamic parameters. Typically, it is derived through a dimensionless transformation from the isotherm model’s equilibrium constant *K_Model_* (L/mol) using

(18) $$K_{Eq}^{0}=\frac{K_{Model}\times C_{Adsorbate}^{0}}{\gamma_{Adsorbate}}$$

where $C_{Adsorbate}^{0}$ is the standard adsorbate’s concentration, typically 1 mol/L, and $\gamma_{Adsorbate}$ is the adsorbate’s activity coefficient, generally as 1.

Many adsorption isotherm models are empirical, resulting in some model constants lacking the physical significance of an equilibrium constant, such as the Freundlich model constant *K_F_*, the Halsey model constant *K_HL_*, and the Brouers–Sotolongo model constant *K_BS_*. Moreover, the dimensions of these model constants incorporate the mass of the adsorbent, complicating their conversion into a thermodynamic equilibrium constant. The concentration of Cr(III) is usually given in mg/L, which often constitutes the model equilibrium constant and can readily be converted to mol/L using the *M_W_* of the adsorbate. Thus, the dimensions of model equilibrium constants for the eight other isotherm models can be converted to a dimensionless equilibrium constant based on the dimension of *K_Model_* [10,39].

Therefore, the model equilibrium constants with units of L/mg, such as *K_L_* in the Langmuir model, *K_FS_* of the Fritz–Schlunder model, *i_JS_* of the Jossens model, and *K_VS_* of the Vieth–Sladek model, can be transformed using

(19) $$K_{Eq}^{0}=K_{model}\times 10^{3}\times M_{W}\times \frac{C_{Adsorbate}^{0}}{\gamma_{Adsorbate}}$$

The dimension of the model equilibrium constants depends on the power of the corresponding model parameters *n*, such as *K_KC_*, *a_(RP)_*, and *K_Sips_* in the Koble–Corrigan, Redlich–Peterson, and Sips models, respectively. $K_{Eq}^{0}$ can be calculated using

(20) $$K_{Eq}^{0}=\sqrt[{n}]{K_{model}}\times M_{W}\times 10^{3}\times \frac{C_{Adsorbate}^{0}}{\gamma_{Adsorbate}}$$

The model equilibrium constant *K_RP_* in the Radke–Prausnitz model can be deduced from model parameters $K_{RP}=\frac{a_{RP}}{b_{RP}}$ and further converted to dimensionless form using

(21) $$K_{Eq}^{0}=\sqrt[{\left(1-m\right)}]{K_{RP}}\times M_{W}\times 10^{3}\times \frac{C_{Adsorbate}^{0}}{\gamma_{Adsorbate}}$$

## 4. Conclusions

This work aimed to explore the stability origin of collagen fiber modified by Cr(III) from adsorption thermodynamics based on isotherm models. The optimal isotherm models for correlating the adsorption process were discerned not just based on the error functions (higher *R*^2^, adjusted *R*^2^, and lower *χ*^2^ values) but also considering the consistency with the physical interpretations of the isotherm function. Consequently, nine isotherm models (namely, Brouers–Sotolongo, Sips, Jossens, Redlich–Peterson, Freundlich, Halsey, Fritz–Schluender, Vieth–Sladek, and Langmuir models) were found to aptly describe the adsorption of the Cr(III) species onto heterogeneous collagen fiber. Of these, only the equilibrium constants from six adsorption isotherms were deemed suitable for calculating the thermodynamic parameters (Δ*G*°, Δ*H*°, and Δ*S*°) according to thermodynamic principles. Furthermore, based on the high *R*^2^ of the van’t Hoff equation, the thermodynamic parameters were reasonably obtained from the Fritz–Schluender model and revealed that the adsorption process of Cr(III) onto collagen fiber was driven by an increase in the system’s entropy. The release of bound H_2_O from collagen was further identified as a key factor in endowment stability. This work emphasizes the significance of Δ*S*° besides conventional energy (Δ*H*°) and recognizes the crucial role of bound H_2_O in the stability of leather manufacture, which sheds light on the development of novel technology to make leather. Additionally, this study establishes criteria for the best-fit model with applicability in varied calculations for specific adsorption systems.

## Figures and Tables

**Table 1:** Parameters for the 11 adsorption isotherm models across various solution pH levels and temperatures.

	Unit	pH			pH			pH		
		3.5			4.0			4.5		
		Temperature			Temperature			Temperature		
		25 °C	35 °C	45 °C	25 °C	35 °C	45 °C	25 °C	35 °C	45 °C
*Q*_*max*_	mg/g	14.63	16.88	78.88	19.65	30.34	93.04	31.00	41.17	103.02
1. Langmuir model										
*Q*_*L*_	mg/g	15.5736	16.4671	97.7820	21.9845	32.3725	113.4898	36.3115	44.4526	115.9466
*K*_*L*_	L/mg	0.0334	0.0870	0.0186	0.0249	0.0313	0.0213	0.0187	0.0341	0.0341
*R* ^2^	-	0.9434	0.9156	0.9853	0.9634	0.9598	0.9792	0.9751	0.9687	0.9760
*Adj-R* ^2^	-	0.9353	0.9036	0.9832	0.9582	0.9541	0.9762	0.9716	0.9642	0.9726
*χ* ^2^	-	1.3949	2.3425	14.9666	1.8249	4.7002	29.6171	3.3698	7.5685	42.8851
2. Freundlich model										
*K*_*F*_	${\left(\mathrm{mg}/g)/(\mathrm{mg}/L\right)}^{n_{F}}$	2.2787	4.1842	7.7367	2.5306	4.4278	10.4484	3.1041	6.2129	14.7199
*n*_*F*_	-	0.3375	0.2542	0.4415	0.3736	0.3502	0.4206	0.4200	0.3504	0.3800
*R* ^2^	-	0.9910	0.9930	0.9906	0.9907	0.9964	0.9929	0.9913	0.9913	0.9916
*Adj-R* ^2^	-	0.9897	0.9920	0.9892	0.9894	0.9959	0.9919	0.9900	0.9900	0.9903
*χ* ^2^	-	0.2211	0.1948	9.6026	0.4618	0.4225	10.1384	1.1858	2.1138	15.1076
3. Halsey model										
*K*_*HL*_	${\left(\mathrm{mg}/g)/(\mathrm{mg}/L\right)}^{n_{HL}}$	0.0872	0.0036	0.0097	0.0835	0.0143	0.0038	0.0675	0.0055	0.0008
*n*_*HL*_	-	2.9628	3.9337	2.2648	2.6760	2.8549	2.3772	2.3809	2.8533	2.6313
*R* ^2^	-	0.9910	0.9930	0.9906	0.9907	0.9964	0.9929	0.9913	0.9913	0.9916
*Adj-R* ^2^	-	0.9897	0.9920	0.9892	0.9894	0.9959	0.9919	0.9900	0.9900	0.9903
*χ* ^2^	-	0.2211	0.1948	9.6026	0.4618	0.4225	10.1384	1.1858	2.1138	15.1075
4. Brouers–Sotolongo model										
*Q*_*BS*_	mg/g	25.3768	25.1269	108.2579	29.4429	52.4230	152.0294	46.7217	55.5578	145.9117
*K*_*BS*_	${\left(\mathrm{mg}/g)/(\mathrm{mg}/L\right)}^{\alpha_{BS}}$	0.0779	0.1584	0.0485	0.0670	0.0736	0.0552	0.0494	0.0887	0.0833
*α*_*BS*_	-	0.4328	0.3514	0.6193	0.5061	0.4444	0.5425	0.5593	0.4982	0.5184
*R* ^2^	-	0.9935	0.9971	0.9965	0.9952	0.9988	0.9959	0.9955	0.9978	0.9969
*Adj-R* ^2^	-	0.9914	0.9961	0.9953	0.9936	0.9984	0.9946	0.9940	0.9970	0.9959
*χ* ^2^	-	0.1862	0.0937	4.2077	0.2805	0.1671	6.7728	0.7145	0.6325	6.3839
5. Fritz–Shluender model										
*Q*_*FS*_	mg/g	−1.17 × 10^45^	−4.04 × 10^44^	1.55 × 10^45^	2.04 × 10^45^	−3.25 × 10^44^	3.35 × 10^45^	−1.90 × 10^45^	−7.61 × 10^45^	1.20 × 10^45^
*K*_*FS*_	L/mg	2.2747	4.1859	7.7597	2.5351	4.4209	10.4362	3.0912	6.1900	14.7328
*m*_*FS*_	-	0.6622	0.7459	0.5591	0.6267	0.6495	0.5792	0.5792	0.6489	0.6202
*R* ^2^	-	0.9910	0.9930	0.9906	0.9907	0.9964	0.9929	0.9913	0.9913	0.9916
*Adj-R* ^2^	-	0.9880	0.9906	0.9874	0.9877	0.9952	0.9905	0.9883	0.9883	0.9887
*χ* ^2^	-	0.2579	0.2273	11.2042	0.5388	0.4929	11.8282	1.3837	2.4666	17.6258
6. Jossens model										
*i*_*JS*_	L/g	5.3860	13.0283	6.8210	3.2684	12.6969	22.2457	2.9425	8.2542	26.5524
*j_JS_*	L/mg	2.0077	2.6862	0.4882	0.9403	2.4578	1.6347	0.5863	0.9268	1.2653
*m_JS_*	-	0.6911	0.7731	0.6617	0.6811	0.6772	0.6274	0.6618	0.7143	0.6874
*R* ^2^	-	0.9920	0.9958	0.9942	0.9931	0.9974	0.9946	0.9944	0.9954	0.9961
*Adj-R* ^2^	-	0.9894	0.9944	0.9923	0.9908	0.9965	0.9928	0.9925	0.9939	0.9948
*χ* ^2^	-	0.2293	0.1371	6.8754	0.4031	0.3591	8.9419	0.8864	1.2966	8.1405
7. Koble–Corrigan model										
*A*_*KC*_	$\left(\mathrm{mg}/g\right)\times {\left(L/\mathrm{mg}\right)}^{n_{KC}}$	2.0052	4.0776	5.3229	1.9977	3.8928	8.5429	2.3060	4.9782	12.3130
*K*_*KC*_	${\left(L/\mathrm{mg}\right)}^{n_{KC}}$	0.0486	0.1105	0.0327	0.0440	0.0465	0.0347	0.0324	0.0620	0.0573
*n*_*KC*_	-	0.4377	0.3665	0.6331	0.5163	0.4521	0.5468	0.5736	0.5188	0.5361
*R* ^2^	-	0.9933	0.9970	0.9960	0.9948	0.9987	0.9957	0.9953	0.9974	0.9968
*Adj-R* ^2^	-	0.9911	0.9959	0.9947	0.9931	0.9982	0.9943	0.9937	0.9966	0.9957
*χ* ^2^	-	0.1925	0.0989	4.7479	0.3025	0.1827	7.1497	0.7473	0.7259	6.7105
8. Radke–Prausnitz model										
*a*_*RP*_	L/g	1.21 × 10^45^	−2.41 × 10^45^	1.29 × 10^46^	−8.26 × 10^44^	−1.28 × 10^45^	1.76 × 10^46^	−1.98 × 10^45^	8.2416	4.56 × 10^45^
*b*_*RP*_	${\left(\mathrm{mg}/g)/(\mathrm{mg}/L\right)}^{m_{RP}}$	2.2771	4.1894	7.7306	2.5346	4.4219	10.4164	3.1114	8.9131	14.7149
*m*_*RP*_	-	0.3376	0.2539	0.4416	0.3733	0.3505	0.4212	0.4195	0.2856	0.3800
*R* ^2^	-	0.9910	0.9930	0.9906	0.9907	0.9964	0.9929	0.9913	0.9954	0.9916
*Adj-R* ^2^	-	0.9880	0.9906	0.9874	0.9877	0.9952	0.9905	0.9883	0.9939	0.9887
*χ* ^2^	-	0.2579	0.2273	11.2031	0.5388	0.4929	11.8292	1.3835	1.2966	17.6255
9. Redlich–Peterson model										
*K*_*(RP)*_	L/g	5.3860	13.0283	6.8210	3.2684	12.6969	22.2457	2.9425	8.2542	26.5524
*a*_*(RP)*_	${\left(L/\mathrm{mg}\right)}^{n_{\left(RP\right)}}$	2.0077	2.6862	0.4882	0.9403	2.4578	1.6347	0.5863	0.9268	1.2653
*n*_*(RP)*_	-	0.6911	0.7731	0.6617	0.6811	0.6772	0.6274	0.6618	0.7143	0.6874
*R* ^2^	-	0.9920	0.9958	0.9942	0.9931	0.9974	0.9946	0.9944	0.9954	0.9961
*Adj-R* ^2^	-	0.9894	0.9944	0.9923	0.9908	0.9965	0.9928	0.9925	0.9939	0.9948
*χ* ^2^	-	0.2293	0.1371	6.8754	0.4031	0.3591	8.9419	0.8864	1.2966	8.1405
10. Sips model										
*Q*_*sips*_	mg/g	41.2450	36.8928	162.5875	45.4424	83.6429	246.3362	71.2752	80.2749	214.9686
*K*_*sips*_	${\left(L/\mathrm{mg}\right)}^{n_{Sips}}$	0.0486	0.1105	0.0327	0.0440	0.0465	0.0347	0.0324	0.0620	0.0573
*n*_*sips*_	-	0.4377	0.3665	0.6331	0.5163	0.4521	0.5468	0.5736	0.5188	0.5360
*R* ^2^	-	0.9933	0.9970	0.9960	0.9948	0.9987	0.9957	0.9953	0.9974	0.9968
*Adj-R* ^2^	-	0.9911	0.9959	0.9947	0.9931	0.9982	0.9943	0.9937	0.9966	0.9957
*χ* ^2^	-	0.1925	0.0989	4.7479	0.3025	0.1827	7.1497	0.7473	0.7259	6.7105
11. Vieth–Sladek model										
*Q*_*VS*_	mg/g	9.2344	11.3718	58.1386	12.8445	18.9478	56.9334	19.5955	28.0660	66.2991
*K*_*VS*_	L/g	0.0228	0.0231	0.1309	0.0300	0.0490	0.2125	0.0513	0.0618	0.2266
*β*_*VS*_	L/mg	0.1154	0.2619	0.0456	0.0785	0.1093	0.0864	0.0609	0.1004	0.1261
*R* ^2^	-	0.9777	0.9743	0.9889	0.9821	0.9864	0.9854	0.9886	0.9863	0.9881
*Adj-R* ^2^	-	0.9703	0.9657	0.9852	0.9762	0.9819	0.9806	0.9848	0.9818	0.9841
*χ* ^2^	-	0.6405	0.8339	13.2171	1.0386	1.8516	24.2260	1.8071	3.8488	24.8572

**Table 2:** Thermodynamic parameters calculated from $K_{Eq}^{0}$ of various adsorption isotherm models.

*T* (K)	pH	*K_Model_*	$K_{Eq}^{0}$	Van’t Hoff Equation	∆*G*°	∆*H*°	∆*S*°
					(kJ·mol^−1^)	(kJ·mol^−1^)	(J·mol^−1^·K^−1^)
1. Langmuir model							
298	3.5	0.0334	1738.88	y = 2619.09x − 0.92 R^2^ =0.12632	−18.49	−21.78	−11.06
308	3.5	0.0870	4521.92		−21.55		−0.74
318	3.5	0.0186	968.24		−18.18		−11.33
298	4.0	0.0249	1295.32	y = 727.10x + 4.83 R^2^ = 0.1618	−17.76	−6.05	39.28
308	4.0	0.0313	1625.00		−18.93		41.82
318	4.0	0.0213	1107.08		−18.53		39.25
298	4.5	0.0187	972.92	y = −2872.68x + 16.61 R^2^ = 0.7661	−17.05	23.88	137.34
308	4.5	0.0341	1774.24		−19.16		139.73
318	4.5	0.0341	1775.28		−19.78		137.30
2. Fritz–Shluender model							
298	3.5	2.2747	1.18 × 10^5^	y = −5825.44x + 31.22 R^2^ = 0.9995	−28.94	48.43	259.63
308	3.5	4.1859	2.18 × 10^5^		−31.47		259.43
318	3.5	7.7597	4.04 × 10^5^		−34.13		259.61
298	4.0	2.5351	1.32 × 10^5^	y = −6663.68x + 34.10 R^2^ = 0.9814	−29.21	55.40	283.92
308	4.0	4.4209	2.30 × 10^5^		−31.61		282.51
318	4.0	10.4362	5.43 × 10^5^		−34.91		283.99
298	4.5	3.0912	1.61 × 10^5^	y = −7379.79x + 36.72 R^2^ = 0.9928	−29.70	61.36	305.57
308	4.5	6.1900	3.22 × 10^5^		−32.47		304.66
318	4.5	14.7328	7.66 × 10^5^		−35.82		305.60
3. Jossens model							
298	3.5	5.3860	2.80 × 10^5^	y = −1215.69x + 16.87 R^2^ = 0.0776	−31.08	10.11	138.21
308	3.5	13.0283	6.77 × 10^5^		−34.38		144.45
318	3.5	6.8210	3.55 × 10^5^		−33.79		138.04
298	4.0	3.2684	1.70 × 10^5^	y = −9135.17x + 42.81 R^2^ = 0.9535	−29.84	75.95	354.99
308	4.0	12.6969	6.60 × 10^5^		−34.31		358.00
318	4.0	22.2457	1.16 × 10^6^		−36.91		354.91
298	4.5	2.9425	1.53 × 10^5^	y = −10413.24x + 36.72 R^2^ = 0.9969	−29.58	86.58	389.79
308	4.5	8.2542	4.29 × 10^5^		−33.21		388.93
318	4.5	26.5524	1.38 × 10^6^		−37.38		389.81
4. Redlich–Peterson model							
298	3.5	2.0077	1.43 × 10^5^	y = 9764.62x − 20.46 R^2^ = 0.6366	−29.40	−81.18	−173.76
308	3.5	2.6862	1.87 × 10^5^		−31.08		−162.66
318	3.5	0.4882	1.76 × 10^4^		−25.84		−174.03
298	4.0	0.9403	4.75 × 10^4^	y = −4221.75x + 25.25 R^2^ = 0.38729	−26.68	35.10	207.32
308	4.0	2.4578	1.96 × 10^5^		−31.21		215.29
318	4.0	1.6347	1.14 × 10^5^		−30.78		207.17
298	4.5	0.5863	2.32 × 10^4^	y = −5459.84x + 28.41 R^2^ = 0.9888	−24.90	45.39	235.87
308	4.5	0.9268	4.67 × 10^4^		−27.53		236.75
318	4.5	1.2653	7.32 × 10^4^		−29.61		235.85
5. Sips model							
298	3.5	0.0486	51.97	y = −7167.05x + 28.04 R^2^ = 0.9916	−9.79	59.59	232.82
308	3.5	0.1105	127.63		−12.42		233.80
318	3.5	0.0327	234.63		−14.43		232.77
298	4.0	0.0440	122.42	y = 544.37x + 2.76 R^2^ = 0.0205	−11.91	−4.53	24.77
308	4.0	0.0465	58.83		−10.43		19.16
318	4.0	0.0347	111.15		−12.45		24.91
298	4.5	0.0324	131.20	y = −3062.14x + 15.25 R^2^ = 0.7889	−12.08	25.46	125.97
308	4.5	0.0620	244.54		−14.08		128.38
318	4.5	0.0573	250.62		−14.60		125.97
6. Vieth–Sladek model							
298	3.5	0.1154	5998.20	y = 4273.29x − 5.22 R^2^ = 0.2653	−21.55	−35.53	−46.90
308	3.5	0.2619	1.36 × 10^4^		−24.38		−36.22
318	3.5	0.0456	2369.64		−20.54		−47.13
298	4.0	0.0785	4084.08	y = −503.38x + 10.09 R^2^ = 0.0924	−20.60	4.19	83.19
308	4.0	0.1093	5684.64		−22.14		85.48
318	4.0	0.0864	4490.72		−22.23		83.09
298	4.5	0.0609	3166.80	y = −3471.52x + 19.75 R^2^ = 0.9637	−19.97	28.86	163.86
308	4.5	0.1004	5221.84		−21.92		164.87
318	4.5	0.1261	6557.72		−23.24		163.82

## Data Availability

The study reported in the article did not use any data.

## Supplementary Materials

The following supporting information can be downloaded at: https://www.mdpi.com/article/10.3390/molecules29020300/s1, Figure S1: Kinetics of Cr(III) adsorption on collagen fibers ([Cr(III)] = 100 mg/L, [collagen fibers] = 0.05 g); Figure S2: Cr(III) species at various solution pH using Visual MINTEQ 3.1, t = 25 °C, [Cr(III)] = 50 mg/L. (Visual MINTEQ is obtained from https://vminteq.lwr.kth.se, accessed on 11 December 2023).
