# Supplementary material for: An Investigation into the Stability Source of Collagen Fiber Modified Using Cr(III): An Adsorption Isotherm Study
Source: Molecules. 2024 Jan 6;29(2):300. doi: 10.3390/molecules29020300 (PMC10818350; doi:10.3390/molecules29020300)
Supplement: Supplementary file 1 [file molecules-29-00300-s001.zip › molecules-2794799-supplementary.pdf]

Supplementary Material

An investigation into the stability source of collagen fiber modified by Cr(III): An adsorption isotherm study

Jiheng Li[a], Wenjun Long[b], Liangqiong Peng[a], Lijun Guo[b], Wenhua Zhang[a,b,*]

a: The Key Laboratory of Leather Chemistry and Engineering of Ministry of Education, Sichuan University, Chengdu 610065, Sichuan, China

b: National Engineering Laboratory for Clean Technology of Leather Manufacture, Sichuan University, Chengdu 610065, Sichuan, China

*: Corresponding Author. The Key Laboratory of Leather Chemistry and Engineering of Ministry of Education, Sichuan University, No.24 South Section 1, Yihuan Road, Chengdu 610065, Sichuan, China.

E-mail address: zhangwh@scu.edu.cn (W. Zhang).

Total pages: 2

Total figures: 2

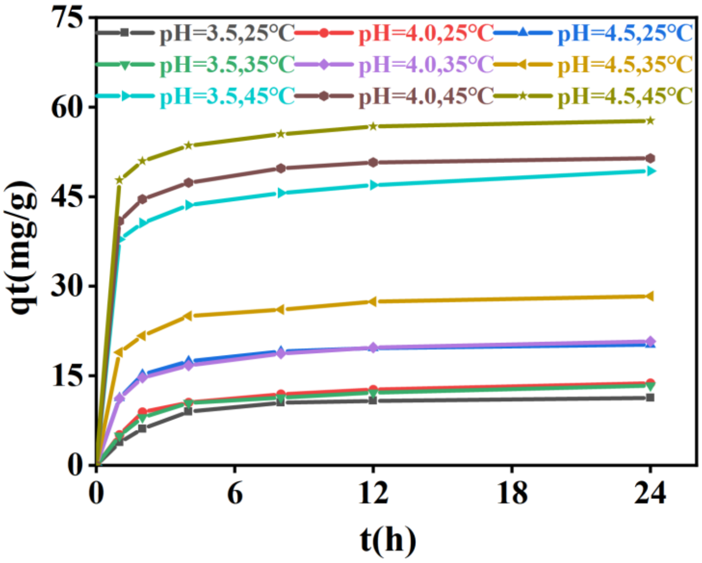

Figure S1 Kinetics of Cr(III) adsorption on collagen fibers ([Cr(III)] = 100mg/L, [collagen fibers] =0.05g).

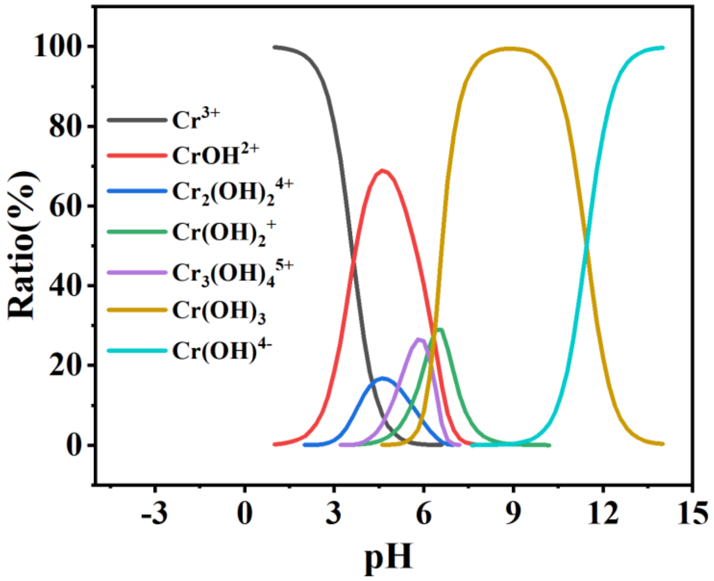

Figure S2 Cr(III) species at various solution pH using *Visual MINTEQ 3.1*, t=25 °C, [Cr(III)]=50 mg/L. (*Visual MINTEQ* is obtained from https://vminteq.lwr.kth.se).
